# Spatial functional mapping of hypoxia inducible factor heterodimerisation and immune checkpoint regulators in clear cell renal cell carcinoma

**DOI:** 10.1038/s44276-023-00033-7

**Published:** 2024-02-09

**Authors:** Elena Safrygina, Christopher Applebee, Alan McIntyre, Julian Padget, Banafshé Larijani

**Affiliations:** 1 Cell Biophysics Laboratory, Centre for Therapeutic Innovation, Life Science Department, University of Bath, Claverton Down, Bath, BA2 7AY UK; 2 ART-AI, Department of Computational Sciences, University of Bath, Claverton Down, Bath, BA2 7AY UK; 3 Centre for Cancer Sciences, School of Medicine, Biodiscovery Institute, Science Road, University of Nottingham, NG7 2RD Nottingham, UK

## Abstract

**Background:**

Clear cell renal cell carcinoma (ccRCC) is a highly malignant subtype of kidney cancer. Ninety percent of ccRCC have inactivating mutations of VHL that stabilise transcription factors, HIF1α and HIF2α, only stabilised in hypoxia. The varied response to HIF2 inhibition, in the preclinical and clinical settings, suggests that assessment of HIF2α activation state, not just expression levels is required as a biomarker of sensitivity to enable optimal clinical use.

**Methods:**

Two-site amplified time-resolved Förster Resonance Energy Transfer (aiFRET), with FRET-Efficiency, $Ef$, as its read out, provides functional proteomics quantification, a precise step forward from protein expression as a tool for patient stratification.

To enhance the clinical accessibility of $Ef$, we have devised a new computational approach, Functional Oncology map (FuncOmap).

**Results:**

FuncOmap directly maps functional states of oncoproteins and allows functional states quantification at an enhanced spatial resolution. The innovative contributions in FuncOmap are the means to co-analyse and map expressional and functional state images and the enhancement of spatial resolution to facilitate clinical application. We show the spatial interactive states HIF2α and HIF1β in ccRCC patient samples.

**Conclusion:**

FuncOmap can be used to quantify heterogeneity in patient response and improve accurate patient stratification, thus enhancing the power of precision.

## Background

Clear cell renal cell carcinoma (ccRCC) is a highly malignant subtype of kidney cancer that represents 80% of 13,100 kidney cancer diagnosed in the UK each year (CRUK). ccRCC are treated with molecular-targeted therapeutics that inhibit immune checkpoints, tyrosine kinases and HIF2α [1]. Responses are heterogeneous and a lack of adequate biomarkers to identify the right drug for the right patient is preventing precision medicine for these patients leading to treatment with costly, toxic therapeutics that might not work. HIF2α is a key target for ccRCC [2–5], and the FDA recently approved a HIF2α inhibitor, Belzutifan, which has an overall response rate of 49% in Von Hippel-Lindau (VHL) mutant ccRCC [6]. Ninety per cent of ccRCC have inactivating mutations of VHL [2, 3]. VHL mutations stablise transcription factors, HIF1α and HIF2α, that are normally only stabilised in hypoxia [7]. Belzutifan prevents HIF2α from dimerising with HIF1β, thereby preventing HIF2α transcriptional activity [4]. Preclinical studies investigating sensitivity to Belzutifan suggest that activation state and not expression per se is a key determinant of response [5, 8]. The varied response to HIF2α inhibition suggests that assessment of HIF2α activation state (binding to HIF1β), not just expression levels could serve as a biomarker to optimise clinical use [8].

ccRCC patients show improved survival following treatment with immune checkpoint inhibitors (ICI) targeting CTLA-4 and PD-1 (Ipilimumab/Nivolumab) [9, 10]. Combination Immune checkpoint and HIF2α targeting clinical trials are underway (Belzutifan/Pembrolizumab) (clinicaltrials.gov). Indeed, immune checkpoint regulators are modulated differently by HIF2α versus HIF1α [10]. Therefore, mechanistically understanding the engagement of HIF2α with HIF1β and the impact that this has on immune checkpoint interactions is key to understanding appropriate patient selection for therapeutic modalities. Accurate patient stratification remains a key factor for optimising both HIF2α inhibitors and ICI.

Improving patient stratification will unleash the power of precision medicine, identifying those most likely to respond. A key problem is the precise selection of the right treatment for each individual patient. The use of expression levels of oncoproteins rather than their activation/interaction states (how they function and interact with other proteins) in therapy/patient selection is imprecise, because expression levels of proteins poorly correlate with an accurate prognosis, whereas the analysis of functinal states does [11, 12].

Our approach to enhance precision is to use quantitative molecular imaging. We have harnessed a two-site amplified time resolved Förster Resonance Energy Transfer (aFRET-intracellular and iFRET-intercellular-aiFRET) method, which is detected by multiple frequency domain fluorescence lifetime imaging microscopy (mfFLIM). Time-resolved FRET is used to measure molecular distances quantifying protein-protein interactions between 1–10 nm. aiFRET provides spatial functional proteomics quantification resulting in a significant and precise step forward from protein levels as a tool for patient stratification.

However, the exploitation of aiFRET has proven to be difficult in the clinical arena. The readout, until recently for functional states of proteins (interactive states or conformational changes due to posttranslational modifications) has been FRET efficiency ($Ef$), where we have shown heterogeneity of *E*_*f*_ via box and whisker plots. Although the quantification has been very precise, the localisation of the *E*_*f*_ from the box and whisker plots on the tissue sections or individual TMA cores remains obscure.

To overcome this limitation and increase the clinical accessibility of aiFRET in this study, we have devised a new algorithm whereby $Ef$ values can be directly calculated from the lifetime images and mapped on to the expression level (fluorescent) image of the oncoproteins under investigation. Our new methodology, Functional Oncology Map (FuncOmap), not only is a direct spatial mapping of the functional states of oncoproteins but also allows per-pixel determination of these functional states. Here, we show the differences in analyses between determining the functional state variations via the traditional box and whisker plots and the new direct spatial functional state determination via FuncOmap. We show for the first time that we can determine HIF2α and HIF1β interactive states in colorectal single cells and patient ccRCC samples. Furthermore, we show that the immune check point regulators (PD-1/PDL-1) interactive states can be determined via FuncOmap in the same ccRCC TMAs. FuncOmap enhances precision and automatically, via the new algorithm, locates *E*_*f*_ on the expression state of the HIF2α and PD-1 in this specific case study. This enables spatial resolution relationships of protein interactions to be identified. FuncOmap can in principle also be used as a general tool for determining the per-pixel functional states of proteins in any type of pathology and not only oncology.

## Materials and methods

### Antibodies and reagents

Recombinant HIF-2α antibody and HIF-1β antibodies were purchased from Abcam (catalogue numbers ab243861 and ab2771 respectively). Monoclonal antibodies mouse anti-PD-1, rabbit anti-PD-L1 were purchased from Abcam (catalogue numbers ab52587 and ab205921 respectively). AffiniPure F(ab’)_2_ fragment donkey anti-mouse IgG (H + L) and peroxidase AffiniPure F(ab’)_2_ fragment donkey anti-rabbit IgG (H + L) were purchased from Jackson Immuno Research (catalogue numbers 715-006-150 and 711-036-152 respectively). Pierce endogenous peroxidase suppressor, TSA SuperBoost kit and Prolong Glass antifade mount were purchased from Thermo Fisher Scientific (catalogue numbers 35000, B40925 and P36980 respectively). ATTO488 NHS ester, bovine serum albumin and rhodamine B, were purchased from Sigma-Aldrich (catalogue numbers A2153-100G and 234141-10G respectively). LS174T- were purchased from ATCC. They were authenticated by STR. A commercial 24 core tissue microarray (TMA-KD241) was purchased from AMSBIO.

## Methods

### Two-site assay for HIF-1β/HIF-2α in colorectal cell lines (LS174T- ATCC)

The cells were tested for mycoplasma contamination seeded in eight well chambers to a confluence of 20%. They were incubated under normoxic and hypoxic conditions (1% O_2_, 48 h) prior to 4% PFA fixation at room temperature for 15 min. Washed twice with PBS and permeabilised with 0.01% TX-100 for 15 min. Followed by two washes with PBS. They were incubated for an hour at room temperature with 1% BSA (10 mg/ml). For the donor-only slides, they were incubated with either anti-PD-1 (at a dilution of 1:100) or anti-HIF-1β (at a dilution of 1:100). The donor-acceptor slides were treated with the following primary antibodies: αPD-1 (1:100), or αHIF-1β (1:100) and αPD-L1 (1:500), or αHIF-2α (1:100). Primary antibodies were incubated overnight at 4 °C. Samples were washed with 0.02% PBST. The samples were treated with secondary F(ab’)2 fragments. F(ab’)2-ATTO488 (at a dilution of 1:100) was introduced to the donor-only slides. As for the donor-acceptor slides, they received both F(ab’)2-ATTO488 (1:100) and F(ab’)2-HRP (1:200). These samples were incubated, for 2 h in the dark, at room temperature in a humidified container. After the incubation period slides were washed with 0.02% PBST. The donor-only slides were mounted with 1 drop of Prolong Glass antifade mount. The donor-acceptor slides were subjected to tyramide signal amplification (TSA) (see below).

### Two-site assay for HIF-1β/HIF-2α and PD-1/PD-L1

The TMAs underwent antigen retrieval process using the Envision Flex retrieval solution, pH 9. The Dako PT-Link system was utilised, where the slides were heated to 95 °C for 20 min. Using a PAP pen, an aqueous-repelling border was outlined around each tissue fragment. Pierce endogenous peroxidase suppressor was then applied to each specimen, and the slides were left to incubate for 30 min at 21 °C room within a humid-controlled environment.

The samples underwent two washes with PBS before being incubated for an hour at room temperature with 3% BSA (10 mg/ml). For the donor-only slides, they were incubated with either anti-PD-1 (at a dilution of 1:100) or anti-HIF-1β (at a dilution of 1:100). The donor-acceptor slides were treated with the following primary antibodies: αPD-1 (1:100), or αHIF-1β (1:100) and αPD-L1 (1:500), or αHIF-2α (1:100). Primary antibodies were incubated overnight at 4 °C. Samples were washed with 0.02% PBST. The samples were treated with secondary F(ab’)2 fragments. F(ab’)2-ATTO488 (at a dilution of 1:100) was introduced to the donor-only slides. As for the donor-acceptor slides, they received both F(ab’)2-ATTO488 (1:100) and F(ab’)2-HRP (1:200). These samples were incubated, for 2 h in the dark, at room temperature in a humidified container. After the incubation period slides were washed with 0.02% PBST. The donor-only slides were mounted with 1 drop of Prolong Glass antifade mount.

The donor-acceptor slides were subjected to tyramide signal amplification (TSA). The purpose of tyramide signal amplification to amplify the acceptor labelling, thus increase the signal-to-noise ratio and enhancing resonance energy transfer. This procedure is described in detail in Veeriah et al. [12] and Sanchez–Magraner et al. [11]. The antibodies were labelled with species-specific F(ab’)_2_ fragments which were conjugated to ATTO488 (donor chromophore, used to label the receptor primary antibody) or HRP (used to label the ligand primary antibody). Tyramide signal amplification was used to conjugate the acceptor chromophore (Alexa594) to the HRP labelling the ligand [PCT/EP2018/062719 and PCT/GB14/050715].

### Time-resolved immune FRET (iFRET) determined by frequency-domain FLIM

The quantitative molecular imaging platform utilises a custom made semi-automated frequency-domain FLIM. The first slide (donor only) was excited by a modulated (40 MHz) diode 473 nm laser, and the lifetime of the donor alone recorded. The second slide was excited by the diode modulated 473 nm laser and lifetime of the donor in the presence of the acceptor recorded. The reduction of donor lifetime (caused by resonance energy transfer) due to the presence of the acceptor reports on distances of 1–10 nm and therefore acts as a “chemical ruler” enabling to quantify receptor-ligand and HIF protein interactions.

We identified the coincidence regions where both the donor and acceptor were observed. A total of 10–20 regions of interest (ROIs) were selected within this coincidence regions. Subsequently, the lifetimes, along with their corresponding standard deviations, were automatically obtained and exported to an Excel spreadsheet.

### Photophysical parameters for quantification of protein interactive states

As input the algorithm takes a lifetime image of donor in the presence of acceptor (*τ*_DA_) and a lifetime image of the donor (*τ*_D_), followed by calculation of reduction of *τ*_DA_ compared to *τ*_D_, which is reflected in a metric called FRET-efficiency:

1 $$Ef=[1- < {\tau }_{{{{{{\rm{DA}}}}}}} > / < {\tau }_{{{{{{\rm{D}}}}}}} > ]\times100$$

FRET-efficiency is calculated as an average for each coincident region.

$Ef$ is directly related to the distance between the donor and acceptor fluorophores (Atto488 and Alexa 594) (Eq. (2)), where *r* is the distance between Atto488 and Alexa594 in these experiments. “*R*_0_” the Förster radius in this case is 5.83 nm and it is the distance whereby the transfer efficiency is 50%. A distance of 5.83 nm corresponds to 4% FRET-Efficiency. Therefore, the significant protein–protein (distance between the donor and acceptor) interactions are considered to be above 4%, indicated by the red line in on the box and whiskers plots:

2 $$Ef=\frac{{R}_{0}^{6}}{{R}_{0}^{6}+{r}^{6}}$$

For each region of coincidence in the two-site assay, we computed the mean distance between the donor and the acceptor:

3 $$r=\,\root 6\of{{R}_{0}^{6}\ast \frac{(1-Ef)}{Ef}}$$

The application generates a table that summarises the quantitative characteristics for each coincident region. This includes the label, area, average FRET efficiency, and the mean distance between the donor and acceptor for each region.

Each overlapping region is colour-coded based on a colourmap that reflects a range from 0 to 50% FRET efficiency. This colour scale is used to create a heatmap, which is mapped automatically on the donor expression image.

### Statistics

The regression graphs for comparison of HIF-2α expression levels with FRET-efficiency utilised Spearman regression to determine the *r*_*s*_ and *p* values. To evaluate FRET-efficiencies in two distinct experimental configurations (PD-1/PD-L1 and HIF-1β/HIF-2α), box and whisker plots were employed to visually represent the data from each TMA independently. This approach aimed to facilitate obtaining the graphs and comparison of FRET efficiency distributions among the group of patient samples from the commercial TMA. Following this, the Mann–Whitney *U* test was utilised to statistically analyse and compare the FRET efficiencies between ccRCC samples and the normal tissue. The null hypothesis presumes no significant difference between the two groups, while the alternative hypothesis poses a significant difference. The implementation of the Mann–Whitney *U*-test resulted in a *p* value. This *p* value signifies the likelihood of observing the differences in FRET efficiencies between the ccRCC and the adjacent normal tissue samples.

### Computational analysis of FuncOmap

**Fig. 1:**
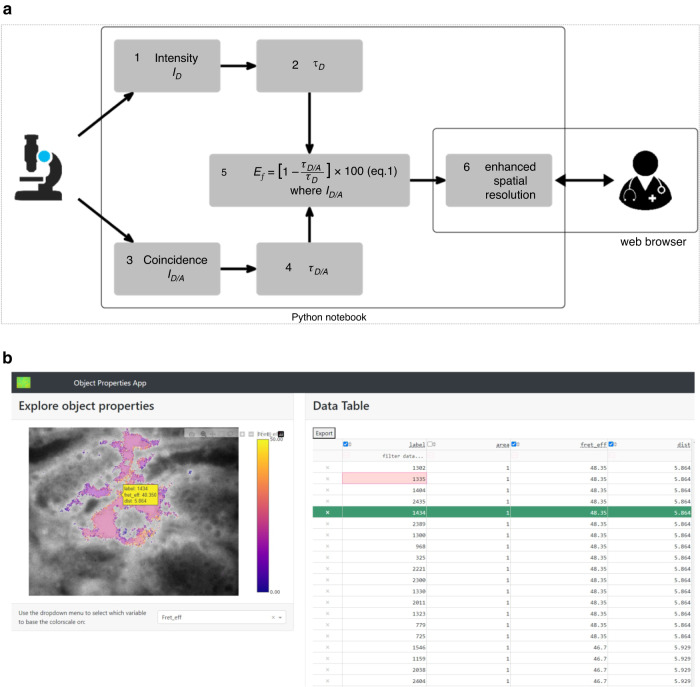
Schematic Illustrating the computational process. **a** Data flow illustrates the progression of information through key stages. Initially, input data fluorescence Intensity (I_D_) and lifetime image t_D_ of the donor as well as the coincident images from the two-site analysis (I _D/A_) and t_D/A_ are stored on Google Drive. This data is retrieved during user execution of the GoogleColaboratory notebook. The notebook execution involves applying functions to process data and retain essential parameters for reproducibility. Finally, running the application enables user interaction with the FuncOmap interface. **b** Interactive FuncOmap in browser. The image on the left is of the coincidence of expressional and functional protein states coloured according to the level of activation as determined by the FRET-efficiency. Below the image, the user can select from a predefined set of thresholds that range from “background” to “3.5 times background,” with “background” set at twice the default value. To the right is a table that is completed to make Table 1.

The initial data are the images captured through a multiple frequency Fluorescence Lifetime Imaging Microscope (mfFLIM). There are two problems to solve from a user perspective: one is how to process that data to provide actionable clinical information, the other is how to enable clinicians to explore the data so they can confirm their diagnosis. We solved the first by constructing a false-colour composite image from the donor and donor/acceptor images which allows the clinician to observe levels of protein expression and protein function on the same image. We solved the second by making the image interactive so that mousing over the image displays additional data ($Ef$) and molecular distance (*r*_nm_), while a drop-down control permits exploration of thresholding to shrink or grow the areas of protein function according to the measured $Ef$. From a computational perspective, the contributions are the choice and composition of the appropriate, validated library software, the construction of the image mapping between expressional and functional states and the spatial enhancement of the coincident image to highlight sites with significant levels of functional activation. The computational process, linked to the corresponding mathematics shown in the equations 1-3, appears in Fig. 1a.

The computational approach outlined in Fig. 1. is a significant advance on the existing method because it is automated—hence reproducible—more precise and provides whole sample functional information. As noted above, the initial data capture provides a donor and a donor-acceptor image because of the optical configuration. For each pixel we record the lifetime in the control donor and the donor/acceptor image and hence calculate the FRET efficiency, which provides the data to carry out a thresholding operation across the image and hence identify all the regions where protein function is above noise level. We use the Otsu thresholding method [13] to convert the original grey level expression image into a binary one, where zeros represent the background and ones indicate regions where the proteins exist. To facilitate user exploration of the data, we provide a set of thresholds for user selection. After applying the threshold to the donor and donor/acceptor images, a logical “and” operation of the two pinpoints the coincident region where both proteins are present. Any pixels outside these overlapping regions are set to zero. Consequently, we identify the (image) coordinates of all coincident regions. Each region is assigned a label and the area it encompasses is calculated, represented as the total number of pixels within each specific coincident region. The “right” level for thresholding is an automatic function of the Otsu algorithm, which adapts to the image supplied to identify the best value to differentiate background from signal. This typically works out at a signal level 4× that of the background but results in images where contrast is too low for human visual perception. This is resolved by increasing the contrast through multiplication of the signal up to 3-fold [12]. This factor only serves to assist human assessment of the images and does not affect the $Ef$ or *r*_(nm)_ information in any way.

In contrast, the previous computational approach uses human annotation of the image to identify coincidence, then generates spreadsheets for donor and donor lifetime in the presence of acceptor. From these changes in lifetime the FRET efficiencies, $Ef$ were calculated (Eq. (1)) and box and whisker statistical distributions plotted. The box and whisker plots provide an average across all the regions, which hides critical spatial localisation of protein activation states. Thus, the drawbacks here are the use of time-consuming human annotation, with attendant inaccuracy (marking regions using a mouse) and incompleteness (some regions that should be marked are not, not least because they can be too small for reliable and consistent identification), the homogenisation of the regions through the analysis process and the inaccessibility (to most clinicians) of a box and whiskers plot as a interpretable diagnostic tool.

The critique above notes that small regions are likely to missed with the manual approach. Small regions remain a problem, in the new approach—not in their detection; the thresholding automatically handles that—but in their presentation to the clinician, since a single pixel, for example, is still too small for ready identification on screen or selection by mouse. For this case, we have introduced a spatial enhancement in the rendering process that aggregates the pixels around one with a high $Ef$, in effect using homogenisation to reveal rather than hide information.

One of the main advantages of the new computational approach is that it allows for a one-step analysis, as opposed to multiple steps. In the new approach the lifetime changes, $Ef$ and thresholding are all processed in an individual pixel. Furthermore, the computational complexity of the new approach is O(n). Each step is linear in the dimensions of the image, since lifetime, FRET efficiency and thresholding all process individual pixels, while the aggregation process for spatial enhancement examines the eight pixels surrounding an individual pixel. The entire process is coded as a Python notebook that generates a web page for the presentation of the protein expression and functional state interactive map (Fig. 1a), from where the clinician can examine the different regions of coincidence by moving a mouse pointer over the image. As illustrated in Fig. 1b, the region selected has a pop-up that shows the data associated with the region and at the same time highlights the row in the data table. The clinician can also explore the effect of choosing different thresholds through the drop-down immediately below the image.

We demonstrate the application of the framework by mapping HIF1β/HIF2α and PD-1/PD-L1 functional states (interaction) states on expression image of HIF1β and PD-1, respectively. The dataset is partitioned into two primary directories, one for the PD-1/PD-L1 experiment and the other for the HIF1β/HIF2α experiment. Within each of these primary directories, seven separate sub-directories exist for each TMA: A1, A2, A5, B1, B5, and B7. These sub-directories contain image files, categorised into four types: Donor Expression, Donor Expression when an Acceptor is present, Donor Lifetime, and Donor Lifetime in the presence of an Acceptor.

FuncOmap allows for the visualisation of protein interaction states through a heatmap representation of average $Ef$ within each coincidence region. By hovering over the heatmap, users can observe the $Ef$ and distance *r*_(nm)_ between proteins in each region of interest (ROI). Additionally, this information can be shown as a spreadsheet, alongside the image, in the browser. Since FuncOmap is implemented on Google Colab, there is no requirement for additional software setup. Google Colab provides a certain amount of memory at no charge and all calculations are performed using cloud-based computational resources.

## Results

### Patient samples

A commercial 24 core tissue microarray (TMA) including samples of ccRCC, and their matched adjacent normal renal tissue was utilised. The normal tissue (B7) was used as a control and the HIF1β/HIF2α and PD-1/PD-L1 activation states were compared to five different ccRCC patients (B5, A5, A2, A1 and B1). The patients B5, A5, A2 and A1 were Stage I and B1 was identified as Stage II. These samples were from male patients with a median age of 65 (range 42–77).

### aFRET quantifies HIF1β/HIF2α quantifies interaction HIFs in normaxic and hypoxic conditions

Prior to determining the interaction of HIF1β/HIF2α in patient samples we validated our approach in single cells (colorectal cells) under conditions of normoxia and hypoxia (1% O_2_, 48 h). Supplementary Fig. 1A shows under normoxic conditions HIF1β and HIF2α do not interact (Median FRET efficiency of 3.95%), whereas there is a significant (*p* = 0.02) interaction of 7.75% (median FRET efficiency) under hypoxic conditions. aFRET efficiency of 4% (see Eqs. 2, 3 in the “Methods”); thus any FRET value below 4% will be regarded as non-interactive. We have also determined whether there is a correlation between the HIF2α expression levels and FRET efficiency. In normoxic conditions there is no correlation. Interestingly, under hypoxic conditions there is a negative correlation with the interactive state. Indicating that lower expression levels of HIF2α correlate with higher interactive states of HIF1β and HIF2α. We demonstrate that high expression levels of HIF2α do not interact strongly with HIF1β (Supplementary Fig. 1B, C). Upon this cellular validation we sought to implement aFRET to patient samples to determine the interactive states of HIF1β/HIF2α in ccRCC patients compared to normal renal tissue.

### aiFRET quantifies HIF1β/HIF2α and PD-1/PD-L1 interactive states in ccRCC patients

**Fig. 2:**
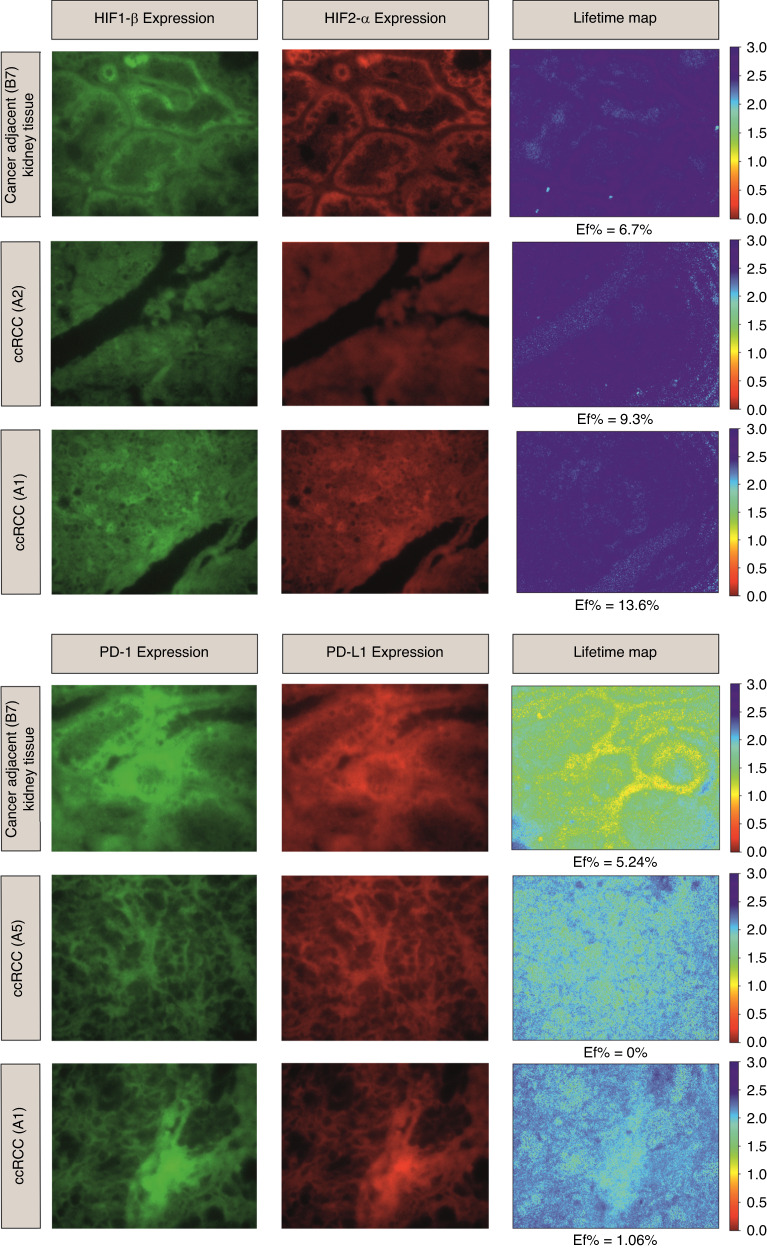
aiFRET quantifies HIF1β/HIF2α and PD-1/PD-L1 interactive states in ccRCC patients. The upper panel  illustrates the expression of HIF1β/HIF2α. HIF1β is the donor and HIF2α is the acceptor. Their lifetime map is shown in the third column with the corresponding calculated median ${{{{{\rm{Ef}}}}}}$. The median ${{{{{\rm{Ef}}}}}}$ for normal tissue was 6.70% and for patients A1 and A2 13.6% and 9.30% respectively. The lower panel shows the expression of PD-1 as the donor and the PDL1 the acceptor. The expression of PD-1/PD-L1 and their average lifetime map with their corresponding calculated median ${{{{{\rm{Ef}}}}}}$ is illustrated. The median ${{{{{\rm{Ef}}}}}}$ for normal tissue was 5.24% and for patients A1 and A2 is 1.06% and 0% respectively.

**Fig. 3:**
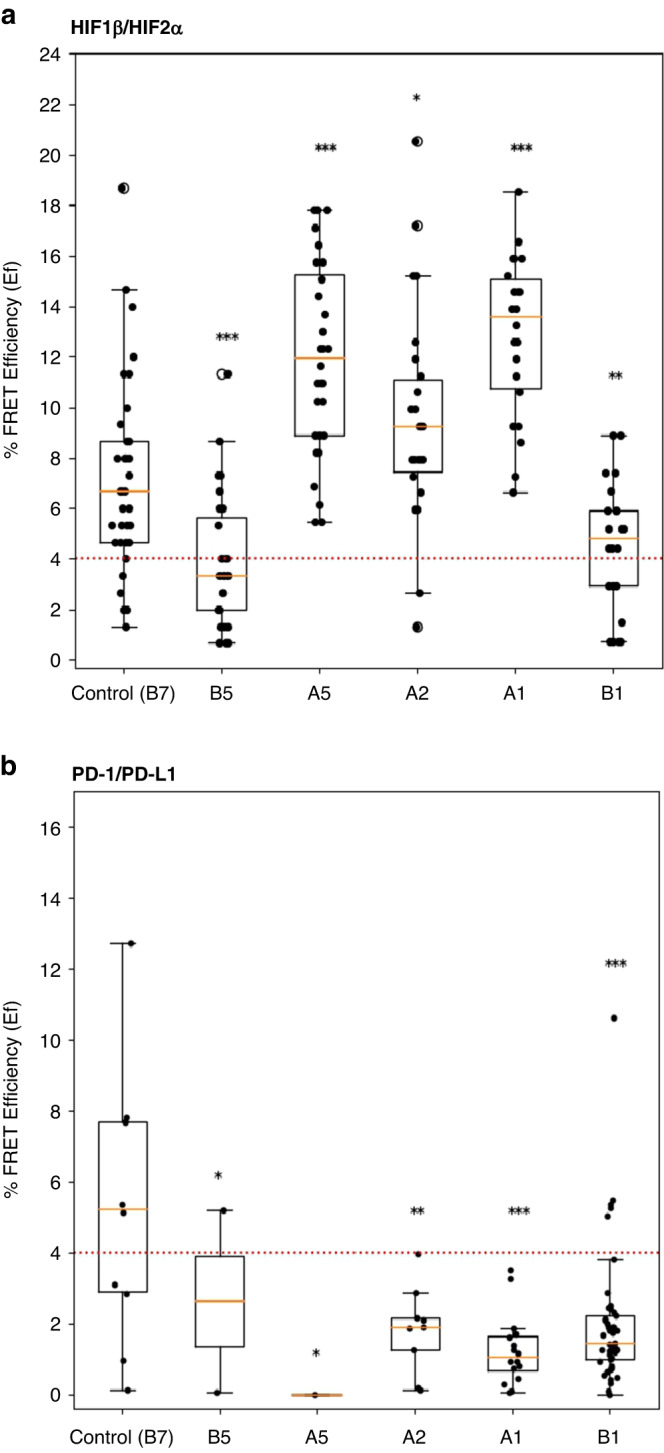
Statistical distribution of HIF1β/HIF2α and PD-1/PD-L1 interactive states in ccRCC patients. **a** Shows the HIF1β/HIF2α box and whisker plots for each patient where the ${{{{{\rm{Ef}}}}}}$ distributions of HIF1β/HIF2α were quantified for each core. **b** Demonstrates the corresponding box and whisker plots for each patient where the ${{{{{\rm{Ef}}}}}}$ distributions of PD-1/PD-L1 were quantified for each core. **a** HIF1β/HIF The exact p values for each tumour core are: B5- *p* = 1.8 × 10^−4^, A5- *p* = 1.4 × 10^−6^, A2- *p* = 3 × 10^−2^, A1- *p* = 1.2 × 10^−6^, B1- *p* = 6 × 10^-3^ (**b**) PD-1/PD-L1 the p values for each tumour core are:B5- *p* = 3.6 × 10^−1^, A5- *p* = 1.8 × 10^−1^, A2- *p* = 3.5 × 10^−2^, A1- *p* = 3 × 10^−3^, B1- *p* = 5 × 10^−3^. *p value of $\ge$5 × 10^−2^, **p value in a range of 5 × 10^−2^ to 5 × 10^−3^, ***p value $\le$5 × 10^−3^. The points on each of the box and whisker plots in A and B correspond to distinct regions of interest (ROI) within each core.

Figure 2 shows the expression of (a) HIF1β/HIF2α and (b) PD-1/PD-L1. These are representative regions of interest from three different ccRCC patients. Two slides were used, one donor-only and one donor-acceptor. HIF1β was assessed on donor only and the second TMA slide was labelled with HIF1β/HIF2α, with HIF2α being the acceptor. Figure 2 (upper panel) illustrates the expression of HIF1β/HIF2α and their lifetime map with the corresponding calculated median $Ef$. The median $Ef$ for normal tissue was 6.70% and for patients A1 and A2 is 13.6% and 9.30%, respectively. Figure 3a shows the corresponding box and whisker plots for each patient where the *E*_*f*_ distributions of HIF1β/HIF2α were quantified for each core.

PD-1/PD-L1 interactive states are shown in Fig. 2 (lower panel), where PD-1 is the donor and the PDL1 the acceptor. The expression of PD-1/PD-L1 and their average lifetime map with their corresponding calculated median $Ef$ is illustrated in Fig. 3b. The median $Ef$ for normal tissue was 5.24% and for patients A1 and A2 is 1.06% and 0%, respectively.

The points on each of the box and whisker plots in Fig. 3a, b correspond to distinct regions of interest (ROI) within each core. Apart from the normal tissue in this incidence the patient samples with a FRET-Efficiency below 4% do not have an interactive state. As noted in the Introduction, the ROIs that indicate the interactive states of both HIF1β/HIF2α and PD-1/PD-L1 cannot be mapped easily on the expression images of the HIF1β and PD-1. Therefore, to directly map the localisation of the interactive states, via $Ef$ calculations, we devised another methodology and implemented this concept to provide location mapped $Ef$ data.

### FuncOmap directly maps the interactive states of HIF1β/HIF2α and PD-1/PD-L1 on the expression images of HIF1β and PD-1

**Fig. 4:**
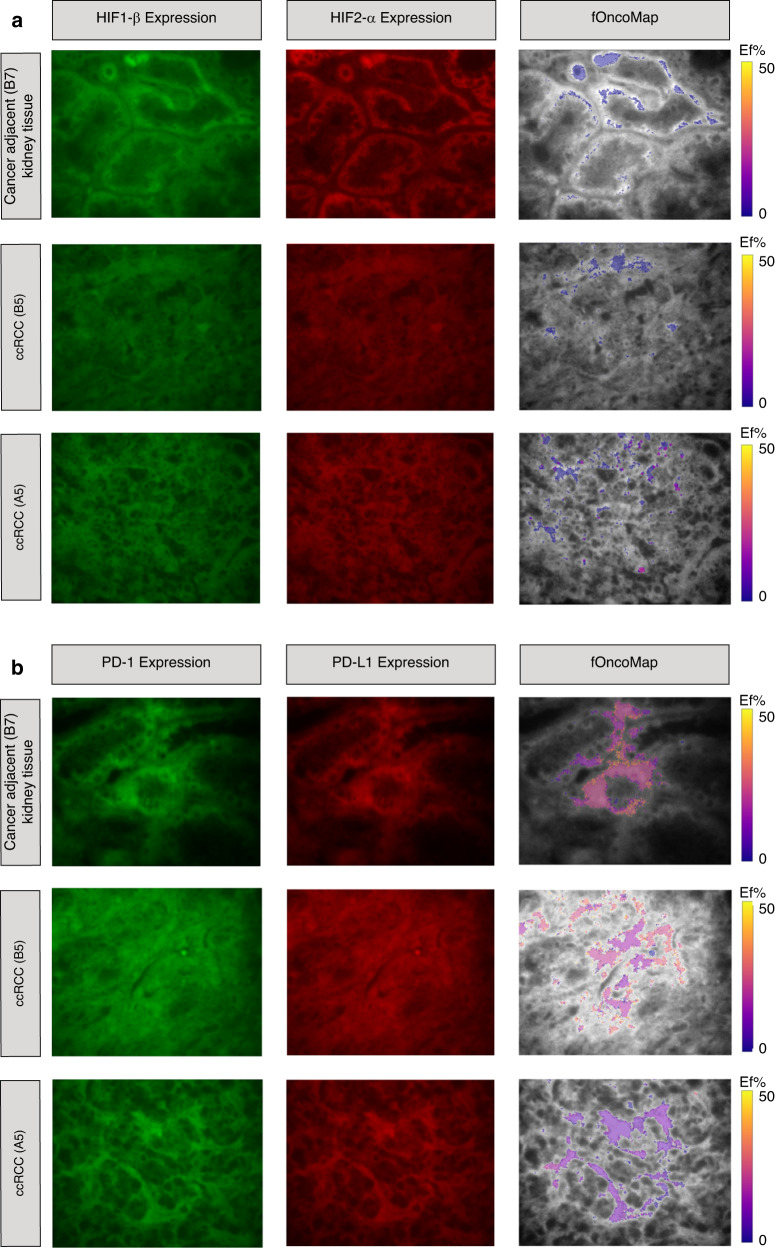
FuncOmap directly maps the interactive states of HIF1β/HIF2α and PD-1/PD-L1 on the expression images of HIF1β and PD-1. **a** Shows the expression of HIF1β/HIF2α and (**b**) PD-1/PD-L1. The two-site assay illustrates regions of coincidence between HIF1β/HIF2α in the control cancer adjacent normal kidney tissue (B7) and 2 additional ccRCC samples A5 and B5. The same cores were labelled for PD-1/PD-L1. The right-hand column of (**a**, **b**) illustrate FuncOmap. The pseudo-colour scale of the ${{{{{\rm{Ef}}}}}}$ ranges from 0.00% (purple) to 50% (yellow).

Figure 4 shows the expression of HIF1β/HIF2α and PD-1/PD-L1. The two-site assay illustrates regions of coincidence between HIF1β/HIF2α in the control cancer adjacent normal kidney tissue (B7) and two additional ccRCC samples A5 and B5. The same cores were labelled for PD-1/PD-L1. The right-hand column of Fig. 4a, b illustrates FuncOmap. The pseudo-colour scale of the *Ef* ranges from 0.00% (purple) to 50% (yellow). FRET efficiency can only have a maximum value of 50% as the Förster radius, *R*_0_, between the donor and acceptor fluorophores (Atto 488 and Alexa 594, respectively) is a constant value of 5.83 nm [14]. Equation (2) in M&M shows that protein-interaction distances, calculated from $Ef$ only have a meaningful value between 5.83 to 10.00 nm (Table 1).

**Table 1:** The quantifiable parameters obtained from regions of coincidence (two-site assay).

1A								
B7			B5			A5		
Coincidence region	Ef(%)	rnm	Coincidence region	Ef(%)	rnm	Coincidence region	Ef(%)	rnm
**662**	7.66	8.78	**61**	0.00		**1307**	21.10	7.23
**672**	4.95	9.49	**103**	0.00		**1302**	19.83	7.32
**685**	0.00		**140**	0.00		**383**	16.03	7.64
**686**	0.00		**230**	0.00		**829**	16.03	7.64
**729**	0.00		**600**	0.00		**189**	16.03	7.64
**402**	0.00		**659**	0.00		**1299**	16.03	7.64
**712**	0.00		**220**	0.00		**235**	15.41	7.70
**950**	0.00		**632**	0.00		**1308**	14.77	7.77
**943**	0.00		**585**	0.00		**78**	13.50	7.90
**657**	0.00		**198**	0.00		**817**	13.50	7.90
**64**	0.00		**174**	0.00		**131**	13.50	7.90
**754**	0.00		**246**	0.00		**294**	13.50	7.90
**664**	0.00		**431**	0.00		**619**	13.50	7.90
**633**	0.00		**586**	0.00		**531**	13.50	7.90
**632**	0.00		**601**	0.00		**83**	12.66	8.00
**719**	0.00		**167**	0.00		**168**	12.24	8.05
**231**	0.00		**318**	0.00		**276**	12.24	8.05
**641**	0.00		**669**	0.00		**400**	12.24	8.05
**635**	0.00		**592**	0.00		**1273**	12.24	8.05
**636**	0.00		**162**	0.00		**826**	12.24	8.05
**637**	0.00		**452**	0.00		**187**	12.24	8.05
**638**	0.00		**80**	0.00		**12**	12.24	8.05
**639**	0.00		**199**	0.00		**822**	12.24	8.05
**640**	0.00		**277**	0.00		**679**	12.24	8.05
**634**	0.00		**242**	0.00		**733**	11.40	8.16

**Table:** 

1B								
B7			B5			A5		
Coincidence region	Ef(%)	rnm	Coincidence region	Ef(%)	rnm	Coincidence region	Ef(%)	rnm
**233**	48.35	5.86	**551**	16.39	7.61	**92**	8.60	8.60
**984**	22.28	7.14	**1104**	21.13	7.22	**63**	10.88	8.23
**1273**	23.41	7.07	**1878**	18.06	7.46	**1250**	10.08	8.35
**24**	21.54	7.19	**1246**	15.76	7.67	**186**	8.16	8.68
**772**	14.25	7.82	**622**	23.15	7.08	**653**	12.09	8.07
**299**	16.28	7.62	**774**	25.15	6.96	**1064**	10.56	8.28
**442**	19.10	7.38	**249**	23.63	7.05	**823**	9.22	8.49
**1932**	26.41	6.88	**1612**	13.92	7.86	**505**	7.72	8.77
**1657**	18.09	7.46	**1288**	26.31	6.89	**110**	8.34	8.65
**384**	16.50	7.60	**586**	16.99	7.56	**639**	11.19	8.19
**648**	26.62	6.87	**1772**	20.77	7.25	**812**	16.69	7.58
**102**	16.10	7.64	**930**	28.31	6.77	**929**	8.25	8.66
**561**	12.82	7.98	**546**	24.76	6.98	**1113**	4.28	9.74
**905**	12.03	8.08	**373**	23.96	7.03	**57**	0.00	
**67**	14.21	7.83	**107**	30.60	6.65	**448**	7.70	8.77
**1750**	18.15	7.46	**1500**	28.54	6.76	**482**	9.31	8.48
**1861**	27.42	6.82	**81**	21.83	7.17	**1274**	8.32	8.65
**548**	23.38	7.07	**1811**	23.98	7.03	**1360**	6.13	9.14
**1009**	28.94	6.74	**1187**	0.00		**1053**	12.95	7.97
**356**	19.97	7.31	**2232**	28.32	6.77	**949**	6.49	9.05
**1973**	28.43	6.76	**176**	28.38	6.77	**1405**	12.38	8.04
**1711**	25.74	6.92	**841**	29.44	6.71	**564**	8.82	8.56
**1919**	14.73	7.77	**261**	15.74	7.67	**1255**	8.24	8.67
**197**	21.18	7.22	**1155**	20.38	7.28	**1241**	6.86	8.96
**1454**	29.85	6.69	**2043**	20.39	7.28	**421**	5.03	9.46

a Shows the coincidence regions where FRET-efficiencies were calculated, corresponding Ef and the related molecular distances for HIF1β/HIF2α.

b Illustrates the coincidence regions where FRET-efficiencies were calculated, corresponding Ef and the related molecular distances for PD-1/PD-L1. It is of note that these values can be directly retrieved from FuncOmap.

FuncOmap shows the pixel distribution of only the coincidence areas between HIF1β/HIF2α and PD-1/PD-L1 in each representative region from the cores. Each pseudo-colour pixel within the coincidence area corresponds to the median $Ef$. Purple pixels/regions signify areas with low interaction to no interaction, while areas coloured in shades of orange and yellow denote regions with the highest interactive states. In the HIF1β/HIF2α samples, the most intense interactive states were apparent in sample A5, while samples B7 and B5 exhibited low or no interaction respectively.

In PD-1/PD-L1 samples, A5 shows the least interaction. The control B7 and B5 exhibited the highest interactive regions. B5 high interaction states were dominant ROIs, whereas in B7, high interactive states were noticeable within specific pixels. This is the first time where the interactive states of PD-1/PD-L1 have been demonstrated in control renal tissue to this accuracy. This may be considered as the homoeostatic interactive states of PD-1/PD-L1 in this type of renal tissue.

FuncOmap directly shows that the functional states do not correlate with the high fluorescent intensities (expression levels of HIF1β and PD-1).

Table 1 presents the coincidence regions where FRET-efficiencies were calculated, corresponding $Ef$ and the related molecular distances. It is of note that these values can be directly retrieved from FuncOmap.

Table 1A shows the FuncOmap for the HIF1α/HIF2β interactions and Table 1B for PD-1/PD-L1. It can be clearly seen that all the molecular distances are greater than *R*_0_ (5.83 nm). The higher the $Ef$ values the closer are the molecular interactions but the valid distances are between 5.83 to 10 nm .

## Discussion

There is an urgent need for predictive biomarkers, particularly in ccRCC, where HIF2α clinical trials and immunotherapy demonstrate an overall response rate of 40–50%. Further to this a 30–50% risk of severe adverse events is identified in ccRCC immunotherapy treated patients [15]. There are no approved genomic- or proteomic-based tools used in the clinic for the precise application of therapies. This is preventing the power of precision medicine from being achieved and produced ongoing problems with patient selection and treatment. Spatial profiling methods, such as immunohistochemistry, proximity ligation assays, Digital Spatial Profiling and single cell RNA sequencing, enhance the complexity of analyses that can be performed and data generated from formalin fixed, paraffin embedded (FFPE) patient samples. These methods can be time consuming, with costly sample preparations and only quantify RNA/protein expression levels and not their structural or post-translational modifications, parameters that are essential regulators of functional behaviour of proteins.

To achieve precision and the correct treatment of patients, posttranslational events and interactive states of proteins need to be quantified [4, 11, 12, 16].

Here we have developed a novel methodology for spatial functional proteomics, FuncOmap. This new direct analysis of functional states spatially maps the interactive events on the expression levels of proteins. This provides data for researchers and clinicians key to clinical decisions and molecular mechanistic understanding about the relationship between expression and interaction and highlights that expression does not correlate with regions with elevated interactive states. The heat map associated with FuncOmap facilitates the interpretation of functional heterogeneity, which is widely accepted as a key feature of therapeutic response, for clinicians and research histopathologists.

Furthermore, by exploiting this quantitative method we have also shown for the first time that we can determine the HIF complex interactive states in single cells under normoxic and hypoxic conditions. Our findings at the single cell level show that high expression levels of HIF2α in hypoxic conditions do not interact strongly with HIF1β. Once again, illustrating that using changes in expression levels of HIF2α is insufficient as a decision-making parameter.

In normal renal tissue and ccRCC patients show the basal level of PD-1/PD-L1 interactive state.

The varied response to HIF2α inhibition in preclinical and clinical studies suggests that an assay of HIF2α activation state (not just expression levels) is required as a more accurate predictive biomarker [8], enabling optimal use of HIF2α inhibitors in the clinic [5]. Of course, other factors including genomic mutations, epigenetic changes, microenvironment may affect clinical outcome.

We have developed a novel biomarker assay which achieves this by quantifying the engagement of HIF2α with HIF1β a determinant of activity [17, 18]. This assay may also be key for longitudinal clinical analysis of patient tumours whilst undergoing treatment with HIF2α inhibitors to detect changes in sensitivity. This is key as mutations in HIF proteins that enable HIF2α and HIF1β heterodimerisation in the presence of HIF2α inhibitors are a mechanism of resistance [19, 20] that could be detected using this biomarker. Further to this a biomarker that accurately quantifies HIF2α-HIF1β interaction could provide a useful tool to determine patients with solid tumours other than ccRCC that frequently exhibit hypoxia and may be sensitive to HIF2α inhibitors. Thus, unlocking of the full potential of HIF2α inhibitors for patient benefit

An interesting finding of this study is that we identify 2/5 patient tumour samples with below normal kidney tissue levels of HIF2α and HIF1β interaction, including one which exhibited no interaction between HIF2α and HIF1β in analysis by FuncOmap. This appears contrary to the widely held view that HIF2α is the main driver of ccRCC [5]. This view is based upon the frequency of VHL mutations, found in >90% of ccRCC which led to HIF2α stabilisation, and the HIF2α functional in vitro investigations using cell lines and murine models of ccRCC which have limitations [21]. An alternative non-transcriptional role for HIF2α could explain the lack of HIF2α and HIF1β interaction in these samples such as the role of HIF2α in protein translation that has been described previously [22]. However, the data generated here suggests that further investigation in a larger sample population of ccRCC tumours is required, using functional proteomics approaches. This is likely key to understanding the inter- and intra- tumour heterogeneity of the role of HIF2α in ccRCC.

There are several advantages of FuncOmap over box and whisker analyses, the main one being that all pixels of the coincident ROIs are considered, rather than calculating an average $Ef$ of the coincident ROI. This provides higher precision in determining the functional states of the oncoproteins under investigation, in this case HIF1α/HIF2β and PD-1/PD-L1. From FuncOmap, the molecular distances of HIF2α/HIF1β and PD-1/PD-L1 can be directly obtained. The knowledge of molecular distances is important for determining mechanisms of drug-targeting. That is the precise variations of the distance (*r*), in Eq. (3), either in protein–protein interactions or changes of protein morphology, can determine whether drugs undergoing clinical trials, have affected the protein’s dysfunctional state. This would be a major asset for determining the pharmacodynamics of newly developed drugs to prevent the high waste of funds with unsuccessful clinical trials.

Routine implementation of FuncOmap with appropriate functional biomarkers in the clinical arena is critically needed to improve overall survival in high-risk patients and significantly reduce the severe adverse events associated with broad use of different therapies.

Our goal is for FuncOmap to be used as a companion diagnostics in clinical trials as well as a generic clinical tool for determining the per-pixel functional states of proteins in any type of pathology.

## Supplementary information

Supplementary Figure 1

Supplementary Figure

## Data Availability

The code for FuncOmap is available under licence for academic research at no cost. For further information, please contact the corresponding authors.

## Code availability

The code is available under licence for academic research at no cost and for commercial use subject to negotiation. For further information, please contact the corresponding authors JP and BL.
