# Correction: Spatial functional mapping of hypoxia inducible factor heterodimerisation and immune checkpoint regulators in clear cell renal cell carcinoma

**DOI:** 10.1038/s44276-024-00052-y

**Published:** 2024-03-26

**Authors:** Elena Safrygina, Christopher Applebee, Alan McIntyre, Julian Padget, Banafshé Larijani

**Affiliations:** 1 Cell Biophysics Laboratory, Centre for Therapeutic Innovation, Life Science Department, University of Bath, Claverton Down, Bath, BA2 7AY UK; 2 ART-AI, Department of Computational Sciences, University of Bath, Claverton Down, Bath, BA2 7AY UK; 3 Centre for Cancer Sciences, School of Medicine, Biodiscovery Institute, Science Road, University of Nottingham, Nottingham, NG7 2RD UK

Correction to: *BJC Reports* 10.1038/s44276-023-00033-7, published online 09 February 2024

In this article, the ‘Data Availability’ statement was corrected from:

“The code for FuncOmap is available under licence for academic research at no cost and for commercial use subject to negotiation. For further information, please contact the corresponding authors.”

to:

“The code for FuncOmap is available under licence for academic research at no cost. For further information, please contact the corresponding authors.”

The original article has been corrected.

