# Supplementary material for: Spatial functional mapping of hypoxia inducible factor heterodimerisation and immune checkpoint regulators in clear cell renal cell carcinoma
Source: BJC Rep. 2024 Feb 9;2:10. doi: 10.1038/s44276-023-00033-7 (PMC11524007; doi:10.1038/s44276-023-00033-7)
Supplement: Supplementary file 1 — Supplementary Figure 1 [file 44276_2023_33_MOESM1_ESM.docx]

**Supplementary Figure 1- aFRET quantifies HIF1β/HIF2α quantifies interaction HIFs in normaxic and hypoxic conditions.**

**(A)** shows under normoxic conditions HIF1β and HIF2α do not interact (Median FRET efficiency of 3.95%) whereas there is a significant (p=0.02) interaction of 7.75% (median FRET efficiency) under hypoxic conditions. a FRET Efficiency of 4%, thus any FRET value below 4% will be regarded as non-interactive.

**(B)** In normoxic conditions there is no correlation between the expression level of HIF2α and FRET-Efficiency.

**(C)** In hypoxic conditions there is a negative correlation with the interactive state. Indicating that lower expression levels of HIF2α correlate with higher interactive states of HIF1β and HIF2α.
