# Supplementary figures and images for: Spatial functional mapping of hypoxia inducible factor heterodimerisation and immune checkpoint regulators in clear cell renal cell carcinoma
Source: BJC Rep. 2024 Feb 9;2:10. doi: 10.1038/s44276-023-00033-7 (PMC11524007; doi:10.1038/s44276-023-00033-7)

## Slide 1
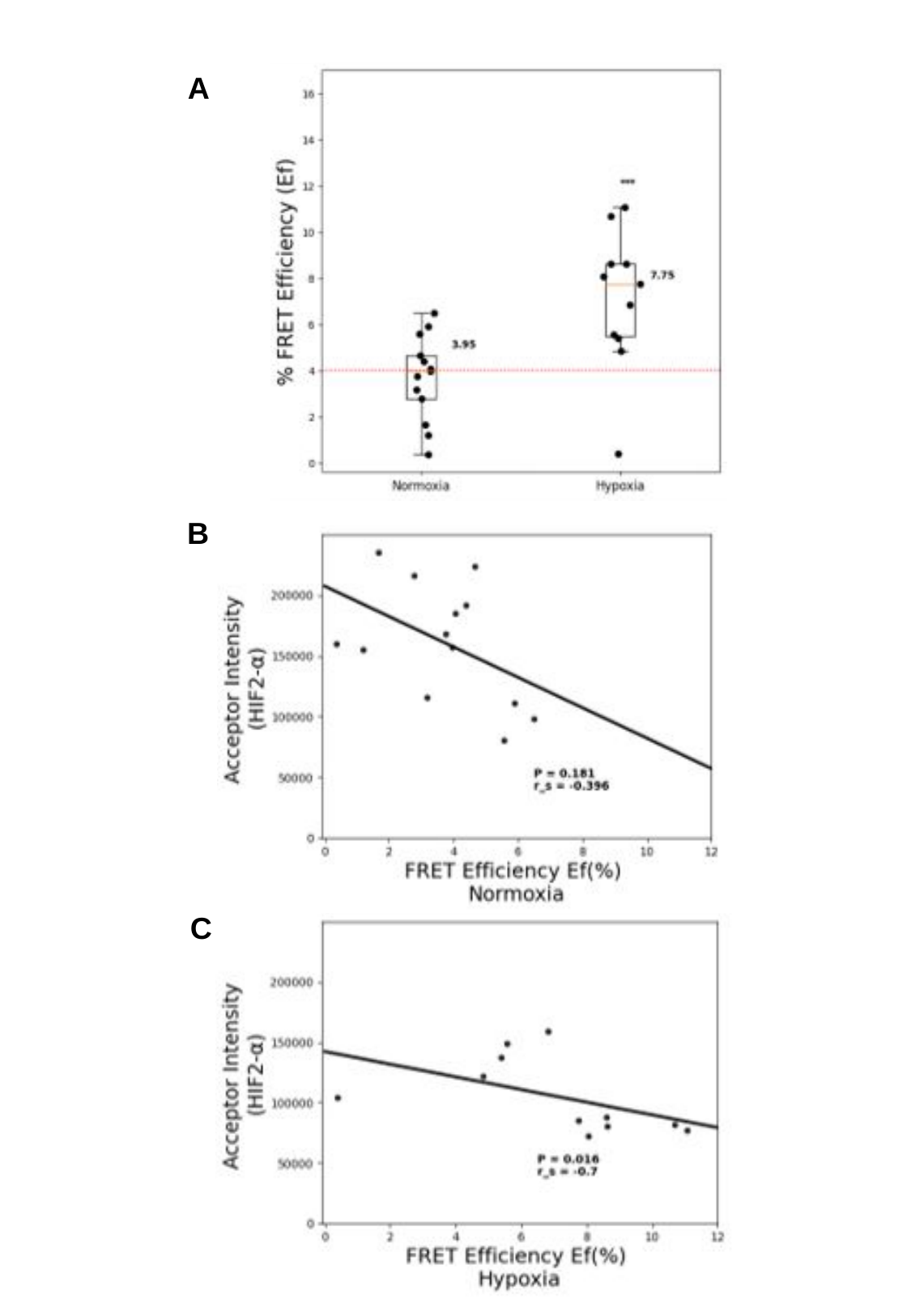

A
B
C

Supplement: Supplementary file 2 — Supplementary Figure [file 44276_2023_33_MOESM2_ESM.pptx]
